# Foodscapes, finance, and faith: Multi-sectoral stakeholder perspectives on the local population health and wellbeing in an urbanizing area in Kenya

**DOI:** 10.3389/fpubh.2022.913851

**Published:** 2022-11-24

**Authors:** Pamela Wadende, Oliver Francis, Rosemary Musuva, Ebele Mogo, Eleanor Turner-Moss, Vincent Were, Charles Obonyo, Louise Foley

**Affiliations:** ^1^School of Education and Human Resource Development, Kisii University, Kisii, Kenya; ^2^MRC Epidemiology Unit, Box 285 Institute of Metabolic Science, Cambridge Biomedical Campus, University of Cambridge, Cambridge, United Kingdom; ^3^Center for Global Health Research, Kenya Medical Research Institute, Kisumu, Kenya

**Keywords:** non-communicable diseases, health, stakeholders, diet, Kenya

## Abstract

**Introduction:**

Rapid urbanization (growth of cities) can upset the local population's health and wellbeing by creating obesogenic environments which increase the burden of non-communicable diseases (NCDs). It is important to understand how stakeholders perceive the impact of urbanizing interventions (such as the construction of a new hypermarket) on the health and wellbeing of local populations. Because low- and middle-income countries (LMICs) lack the reliable infrastructure to mitigate the effects of obesogenic environments, so engaging stakeholders who influence dietary habits is one population-level strategy for reducing the burden of NCDs caused by newly built developments.

**Methods:**

We conducted key informant interviews with 36 stakeholders (25 regulatory and 11 local community stakeholders) from Kisumu and Homa Bay Counties of Western Kenya in June 2019. We collected stakeholders' perspectives on the impacts of a new Mall and supermarket in Kisumu, and existing supermarkets in Homa Bay on the health and wellbeing of local populations.

**Results:**

Through thematic discourse analysis, we noted that some stakeholders thought supermarkets enabled access to unhealthy food items despite these outlets being also reliable food sources for discerning shoppers. Others linked the changing physical environment to both an increase in pollution and different types of diseases. Stakeholders were unsure if the pricing and convenience of supermarkets would stop local populations from buying from their usual small-scale food vendors. The key finding of this study was that engaging relevant stakeholders as part of population health impact assessments of new developments in cities are important as it directs focus on health equity and prevention in instances of resource constraints. The findings highlight, also, that community members have a strong awareness of the potential for interventions that would improve the health and wellbeing of local populations.

## Background

Urbanization has an impact on the health and wellbeing of local populations (1–4). Lack of physical space for energy expenditure activities such as exercise, sedentarization of workplaces, and eating high energy readily available food fueled by the nutritional transition often accompanies urbanization (5, 6). Increased caloric intake and decreased physical activity exercise have fueled obesity and related non-communicable diseases (NCDs). Indeed, the World Health Organization (WHO) in 2021 noted that in 2016 an estimated 650 million adults in the world were obese (7).

The growing burden and cost of managing NCDs on families, communities, and the healthcare delivery system are substantial and have prompted a call for action at global, regional, and national levels (1–4). Dalal et al. (8) note that the prevalence of obesity in sub-Saharan Africa stands at up to 43%, hypertension at up to 48%, and diabetes at up to 16%. Data from demographic and health surveillance systems show increasing trends of NCD morbidity and mortality in sub-Saharan Africa (9–11). Hospital-based studies revealed rising adult admissions and deaths attributable to NCD-related conditions (12–14).

Living in an informal settlement in an urban area increases the risks of hypertension, diabetes, and high cholesterol. A population-based household survey of 5,000 participants in Nakuru town, Western Kenya (15) found a 13% prevalence of obesity, 50% of hypertension, 7% of diabetes, and 21% of high cholesterol. In 2010, a population-based survey in Nairobi's Kibera slum found a 23% prevalence of hypertension and a 5% prevalence of diabetes (16, 17). In the informal settlements/slums Korogocho and Viwandani, Van de Vijver et al. (18) survey on hypertension revealed a 12% prevalence with only 19% awareness of health conditions. These results are similar to those found by Ezzati et al. when looking at the lifestyles and diets of people who had moved from rural to urban areas in LMICs (19). The variation in estimates deserves consideration in and of itself, but even the lower estimates represent a significant burden of NCDs that policymakers understand and prioritize.

There is a nutrition transition being experienced in LMICs, characterized by a move from rural to urban areas and the adoption of lifestyles and diets that increase the risk of NCDs (20, 21) and environmental pollution (22, 23). Although supermarkets play a key role in driving the nutrition transition through increasing the availability of processed food, these same supermarkets also sell healthy food cheaply and can facilitate access to a wider range of foods (24, 25). According to Rischke et al. (24) and Demmler et al. (25), Kenya has a prospering supermarket sector accounting for approximately 10% of the share of grocery sales at the national level and higher in urban centers which increased the consumption of processed food among locals (24, 25) and were associated with a higher body mass index and metabolic syndrome in adults (21, 25, 26) but a lower probability of children being underweight (26). Clearly, with appropriate interventions and support for populations, supermarkets have the potential to improve diet and health by supporting healthier food choices.

In Kenya, it has become apparent that various societal factors, most of which do not fall within the reach of the Ministry of Health (MoH), determine the increase of NCDs. These factors include rapid urbanization and attendant environmental pollution (22, 23). Therefore, sectors including agriculture, education, transport, and faith became important actors in efforts to engage with NCDs through their activities shaping, for example, food production and retail, transport behavior, and emotional, spiritual, and physical health of a population. Arora et al. (27) describe a multi-sector action (MSA) approach as other sectors of the economy working with or without the collaboration of the health sector to achieve health outcomes. The WHO particularly emphasized in the 2011 Moscow declaration, the importance of strengthening the government's role and developing multi-sectoral public policies and legal frameworks for NCD prevention, alongside strengthening health systems. As detailed by Juma et al. (28), Kenya is among other LMICs such as Cameroon, Nigeria, and Malawi that have adopted an MSA approach so as not to leave NCD prevention and control to only the Ministries of Health, which are often challenged by inadequacies of requisite infrastructure and resources.

To explore the multi-sectoral approach to engaging NCDs and overall population health and wellbeing in local communities in Kenya, we conducted focus group discussions and individual interviews as part of a broader natural experimental study (29). We collected the stakeholder's perspectives on the impact of the newly developed Lake Basin Mall in Kisumu and various supermarkets in Homa Bay on the health and wellbeing of local populations. Participants included leaders of business licensing bodies, such as county trade officers, National Environmental Management Authority (NEMA) impact assessment officials, and public health risk assessors. We refer to this group as regulatory stakeholders. The next group we term local community stakeholders are community influencers who have great persuasion power and are thus very likely to successfully advise community members on a wide range of issues, even if they lack formal training in the areas in which they speak. Examples of local community stakeholders include faith groups such as pastors and Imams, village elders, and other local pressure groups such as the local public transport welfare association leaders. This whole study combined quantitative and qualitative research methods to evaluate changes in dietary and health behavior coupled with furthering an understanding of the drivers and impacts of these changes. The study also included household survey data, audits of local foodscapes, and shopper intercept surveys. The baseline data collection took place in 2019, before the anticipated opening of the hypermarket with a follow-up collection of approximately 1 year after the hypermarket opens to allow shopping habits to adjust.

### Study aim and objectives

We collected perceptions of stakeholders on the expected impact of the new Lake Basin Mall in Kisumu (intervention site) and supermarkets in Homa Bay (control site), on local diets and the environment as it affects the health and wellbeing of community members. Our four questions were focused on the following: type and involvement details of stakeholders associated with the development of food retail outlets; if they thought the development would impact local diets, health, wellbeing, and the environment; any predictions about the future of local diets, health, and the environment; and any evidence of a multi-sectoral approach to engaging NCDs.

## Materials and methods

### Study design and setting

This analysis is drawn from the baseline assessment of a natural experimental study conducted in the Mamboleo area of Kisumu (intervention site, population 968909-Kenya National Bureau of Statistics 2019 census data) and Sofia area of Homa Bay Counties (comparison site, Population 963794-Kenya National Bureau of Statistics 2019 census data); both in the Western Kenya region. The Mamboleo area in Kisumu is the intervention site that has an upcoming hypermarket and the Sofia area in Homa Bay is the control site that neither has a similar hypermarket nor any planned for in its future. These areas were delineated using existing spatial census data, field visits, and local knowledge of the study investigators. A 2 km radial buffer was drawn around the hypermarket in Kisumu and matched according to population density with a 2 km radial buffer around the center of Sofia area, Homa Bay County 100 Kilometers away from Kisumu. The two study sites had approximately similar aggregate socioeconomic, topographical, and food retail characteristics. Furthermore, both areas include a mix of lower and higher socio-economic status (SES) areas, both informal and formal settlements; a similar number of supermarkets and major roads. Both areas are located on the shores of Lake Victoria, a freshwater lake and the traditional ancestral lands of the Luo ethnic community; thus, participants share similar cultural practices including dietary preferences. The two marked differences in the characteristics of the sites were that Mamboleo is urban and has a hypermarket, whereas Sofia is peri-urban and does not have a hypermarket. It was thought to be valuable to collect stakeholder perspectives from both areas to understand any differences. Cultural practices around diet have a direct relationship to trends in NCD incidents in local communities. Indeed, according to the KDHS of 2014 (30), approximately 9% of women and 3% of men in Kenya have been told by a health service provider that they have hypertension.

### About Lake Basin mall

The Lake Basin Mall is a hypermarket developed using Kenyan public funds with support from the World Bank, through the Lake Basin Development Authority; a parastatal, (an organization having political authority and serving the state directly) in Kenya. The hypermarket development, with its various planned social service providers, is complete but its official opening is shrouded in controversy; gauging from reports by the Kenyan Ethics and Anti-Corruption Commission (EACC)—that its developers inflated the construction cost by 2.5 billion Kenyan shillings (about 23.3 million USD) (31).

### Theoretical framework

Stakeholders' perspectives can be understood using Urie Bronfenbrenner's socio-ecological model (32) which was later formalized into the socio-ecological theory, widely used in deciphering human action. This model provides a framework for understanding how multiple actors work in concert to impact an individual's behavior. Socio-ecological models are useful in analyzing health behaviors as they categorize factors that influence behaviors on multiple levels; intrapersonal, interpersonal, organizational, environmental, and policy and legislative. Since the models focus on the importance of context in behavior, they also highlight a variety of opportunities for intervention.

### Ethical considerations

This analysis forms part of a wider natural experimental study that was reviewed and approved by the Scientific Ethical Review Unit (SERU) of the Kenya Medical Research Institute (KEMRI, SSC 3730). We obtained permission from the County Ministry of Health and local authorities, opinion leaders, village elders, and community members after listening to us describe study processes. We obtained informed consent obtained from all respondents and deleted personal identifiers from the data set before analysis.

### Participants

Before we conducted the interviews for this study, we held stakeholder engagement meetings in both study sites in November 2018. During these initial meetings, the study staff explained the study objectives and sought input from the stakeholders—including on how the study might be improved and who was best placed to work as data clerks in the community. We purposefully selected stakeholders from the initial engagement meetings and subsequently identified others through a snowballing process.

### Data collection and analysis

We conducted key informant interviews with 36 stakeholders (25 regulatory and 11 local community stakeholders) from Kisumu and Homa Bay Counties of Western Kenya in June 2019. The interviewers were all Kenyan and residents of the local research sites; Homa Bay and Kisumu. They spoke the local language Dholuo, Kenya's National language Kiswahili, and Kenya's official language English; all these languages Kenyans commonly mix in conversation. All interviewers had an undergraduate degree and above and conducted the interviews in pairs of female and male subjects. Interviews took between 45 min and 1 h and followed a prepared guide on which the interviewers had been trained.

The main questions for the interviews in Kisumu sought information about the development of the mall and supermarket and what they thought about the possible impacts on the diets of the catchment community in Kisumu. Other details included what the interviewees thought about the future impact of the Lake Basin Mall—and specifically the hypermarket—on the local diets, economy, way of life, and other effects across the community. All interviews were audio recorded and transcribed verbatim. The interviews were also translated into English as necessary since the responses at the time were a mixture of languages such as Kiswahili and Dholuo (the national and predominant local language, respectively) apart from English.

A total of 10 research assistants (4 for the Kisumu site and 6 for the Homa Bay site) took part in coding the data. We started the coding exercise with an orientation workshop for the coding team. In this workshop, we jointly developed a codebook to guide the coders in the analysis process and each coder took charge and led the analysis of a complete transcript but sought others' opinions whenever they needed clarifications and consensus. Through thematic discourse analysis, we followed a stepped process that began with reading and re-reading the transcribed interviews and coming up with codes. We combined these codes into themes that represented the data and then examined in detail which themes to report. We chose themes worthy of reporting as per our objectives and research questions. The background framework of the socio-ecological model of understanding diet choice as being influenced by determinants located in their environment and consequent relationships guided our inductive process of linking the themes to the data. We observed the three domains under which it is prudent to report qualitative data; research team and reflexivity, study design, and data analysis and findings presented in the 32-item checklist of the consolidated criteria for reporting qualitative research (COREQ) (33). We include a detailed description of how we used this checklist in Appendix I. In this analysis exercise, we were aided by NVIVO version 11 for the Kisumu site and Version 12 for the Homa Bay site. The upgrade did not in any way impact our analysis process so we did not have to make any changes to the plans.

### Findings

We interviewed 25 regulatory and 11 (29 men and seven women all 30+ years) local community stakeholders in both Kisumu and Homa Bay using a similar interview schedule developed by the researchers. The number of stakeholders per site was: regulatory 15 for Kisumu and 10 for Homa Bay; local community five for Kisumu and six for Homa Bay. The main themes are captured in Table 1 and also detailed below with exemplary quotes.

**Table 1:** Summary of main findings.

**Theme**	**Sample quotation**
Health implications of fast foods	“*One thing I know in a hyper supermarket, there are going to be these fast foods, and fast food are not healthy … like French fries, sausages, fried chicken, yeah those are the ones that… those are the ones actually that sell, yeah”* regulatory stakeholder: Kisumu. “*Of course, the sale of these foods often leads to overweight and obesity in children, if it is in adults, it could be related to maybe hypertension if you are overweight, so that can be a negative* [outcome of a new supermarket]*,”* regulatory stakeholder: Kisumu “*…it is about education of our local community, like you know in* [faith community] *we normally encourage our members to practice vegetable diet but you know we normally tell them that they have freedom to choose”* local community stakeholder: Homabay
Supermarkets as reliable, but selective, food outlets	“*You know in the supermarket even the prices of these commodities they are a little bit affordable, you can find that in this small kiosk a loaf of bread is fifty shillings but, in the supermarket, you will get it at forty-seven so I rather walk in supermarket, go buy my loaf of bread which is forty-seven and save three shillings”* local community stakeholder: Homabay “*They will be there still because not, not everybody living in those areas will want to go to the Mall, like if you have maybe a hundred shillings and you want to go into the supermarket or that Mall to buy maybe… tomatoes and you are not sure whether a kilo in that supermarket today as you are going will be a hundred bob, then you opt to buy from outside. But there are people who… don't mind about the cost of what they are going to buy in the supermarket even if it is vegetables or the herbs and spices or fruit. His or hers is to get the fruit he is looking for. So that's why it depends on the categories of people that are there.”* regulatory stakeholder, Kisumu
Perceived mixed impacts on the local community	“*…you will find that now people no longer need to travel far to buy, to get some things because supermarket brought them near, secondly consumption of processed goods … you'll find a lot of processed goods in the supermarket as opposed to organic foods”* regulatory stakeholder: Kisumu “*I don't think there'll be any influence because when I look at the local I'm assuming the… the hyper market has its clientele then the local community also have their clientele that go to the food outlets and the kind of food outlets we have in the community is different from what we are going to have in the mall…*, regulatory stakeholder: Kisumu “*There will be loss of jobs… because many people prefer according to me going to the supermarket than to, to open-air market.”* Local community stakeholder: Kisumu
Traffic and air pollution	“*In terms of pollution of course if you are exposed to contaminants then you might get short-term or long-term diseases…”* regulatory stakeholder: Homabay “*The negative there will be generation of waste, there will be pollution that is both noise, air and water. There might be crime, of course, any place that has development may experience* [incidences of] *prostitution or even theft …those are the negative* [effects]*....”* regulatory stakeholder, Kisumu

### Health implications of fast foods

Both regulatory and local community stakeholders were concerned about the health of the local population, believing that supermarkets make it easier for people to access unhealthy fast food (including potato fries and cured meats) and thus change local diets. Regulatory stakeholders believed that such food they considered unhealthy, results in overweight, obesity in children, and non-communicable diseases such as hypertension in adults. An important contrast to these ideas by regulatory stakeholders is that although local community stakeholders acknowledged that supermarkets could avail unhealthy diets to local populations, they were confident they could share knowledge that helped community members discern healthy from unhealthy food available in the supermarkets. Additionally, these local community stakeholders could appeal to the community members to avoid unhealthy food presented in supermarkets.

“*One thing I know in a hyper supermarket, there are going to be these fast foods, and fast food are not healthy … like French fries, sausages, fried chicken, yeah those are the ones that… those are the ones actually that sell, yeah”* regulatory stakeholder: Kisumu

Stakeholders even went further to pronounce themselves on the health implications of poor diets on local populations.

“*Of course, the sale of these foods often leads to overweight and obesity in children, if it is in adults, it could be related to maybe hypertension if you are overweight, so that can be a negative* [outcome of a new supermarket]*,”* regulatory stakeholder: Kisumu

In contrast to regulatory stakeholders, local community stakeholders such as faith leaders were confident, that they could educate and persuade community members to avoid fast food. They utilized the knowledge they had about the relationship between unhealthy diets and disease to appeal to local populations to choose healthy food. Indeed, they affirmed already giving dietary advice.

“*…it is about education of our local community, like you know in* [faith community] *we normally encourage our members to practice vegetable diet but you know we normally tell them that they have freedom to choose”* local community stakeholder: Homa Bay

### Supermarkets as reliable, but selective, food outlets

There were mixed feelings about the value of supermarkets as food outlets. While local community stakeholders thought supermarkets offered a reliable and dependable shopping experience in terms of prices, availability, and quality of food, having a fixed location as opposed to commonly mobile food hawkers, some regulatory stakeholders thought supermarkets were expensive and thus exclusive. One possibility suggested by stakeholders was that shoppers were able to buy particular items from the supermarket and other from their regular retailers, in local communities, depending on the prices and availability in the different outlets.

“*You know in the supermarket even the prices of these commodities are a little bit affordable, you can find that in this small kiosk a loaf of bread is fifty shillings but, in the supermarket, you will get it at forty-seven so I rather walk in supermarket, go buy my loaf of bread which is forty-seven and save three shillings”* local community stakeholder: Homa Bay

Some stakeholders thought that customers could choose what food items to buy from the hypermarket and what to obtain from the local food retailers, depending on how much they could spend on food at that time.

“*They will be there still because not, not everybody living in those areas will want to go to the Mall, like if you have maybe a hundred shillings and you want to go into the supermarket or that Mall to buy maybe… tomatoes and you are not sure whether a kilo in that supermarket today as you are going will be a hundred bob, then you opt to buy from outside. But there are people who… don't mind about the cost of what they are going to buy in the supermarket even if it is vegetables or the herbs and spices or fruit. His or hers is to get the fruit he is looking for. So that's why it depends on the categories of people that are there.”* regulatory stakeholder: Kisumu

### Perceived mixed impacts on the local community

Both groups of stakeholders related mixed feelings about the impact of the new supermarket on local businesses and local food retailers. Variously, they thought that the presence of the mall and supermarket in the local community was both beneficial and injurious to the local population and food retail businesses in the community. Regulatory stakeholders thought that supermarkets brought certain food close to where people lived but most of these would be unhealthy processed food as opposed to organic foods. They acknowledged, however, that the supermarket and other retail outlets have their dedicated clientele. Local community stakeholders went further and reflected on the impact of the new Mall on local families which included causing job losses among local food retailers when people start buying food from the supermarkets instead.

“*…you will find that now people no longer need to travel far to buy, to get some things because supermarket brought them near, secondly consumption of processed goods … you'll find a lot of processed goods in the supermarket as opposed to organic foods”* regulatory stakeholder: Kisumu

Some stakeholders believed that both the hypermarket and the local food retailers had their own unique clients and so none could take the other's regular clients.

“*I don't think there'll be any influence because when I look at the local I'm assuming the… the hyper market has its clientele then the local community also have their clientele that go to the food outlets and the kind of food outlets we have in the community is different from what we are going to have in the mall…,”* regulatory stakeholder: Kisumu

A local community stakeholder reflected on the impacts of new supermarkets at the family level when it caused job losses as local food retailers lose customers to supermarkets that would not employ as many people as local retailers do. Such a scenario would directly impact the family.

“*There will be loss of jobs… because many people prefer according to me going to the supermarket than to the open-air market.”* local community stakeholder: Kisumu

### One stakeholder thought the hypermarket would not employ as many people as the small retailers already did

“*...it may not employ as many people as small traders and at the same time it may kill those businesses of smaller traders”* regulatory stakeholder: Homa Bay

However, a regulatory stakeholder thought that the new mall and supermarket would generate jobs for the local community such as in transport, banking, and retail.

“*…Then for some, some would benefit… if you look at the example of the* [transport business] *people, they are the major people who ferry people to the supermarket and away from the supermarket so for them I think the supermarket has helped them in terms of increasing their business opportunities…Then maybe things like the banks also...”* regulatory stakeholder: Kisumu

### Traffic and air pollution

On environmental pollution, both regulatory and local community stakeholders were in agreement that the Mall would increase economic activities which would be evidenced by increased vehicular movement around it resulting in harmful air, water, and noise pollution. Such pollution would increase incidents of diseases experienced by people in the local community. The increased vehicular traffic was also seen by the regulatory stakeholders to increase the incidents of crime in the local area. This will occur when crimes such as prostitution and theft increase due to increased economic activities.

“*In terms of pollution of course if you are exposed to contaminants then you might get short-term or long-term diseases…”* regulatory stakeholder: Homa Bay

### On issues to do with increased waste generation by the hypermarket, a stakeholder had this to say

“*The negative effects will be a generation of waste, there will be pollution of noise, air, and water. There might be a crime, of course, any place that has development may experience* [incidences of] *prostitution or even theft …those are the negative* [effects]*....”* regulatory stakeholder: Kisumu

## Discussion

### Key findings

The study questions focused on the type and role of the influence of stakeholders in the development of food retail outlets, their views and predictions on the outlet's impact on local diets and food retail, population health, and wellbeing, and evidence of a multisectoral approach in their perspectives.

Our data showed that both the regulatory and local community stakeholders had an influence on the development of the mall and supermarkets in various ways. Although the regulatory stakeholders were important in licensing food retail outlets, the local community stakeholders both openly and, at times, tacitly persuaded the local population to buy from, or not to, the food retail outlets and thus were important in deciding the location of these outlets.

Regarding the impact of the mall and supermarkets on local diets and food retail, stakeholders had mixed responses. They were in agreement that the mall would impact local diets in both positive and negative ways; such as enabling access to both healthy and unhealthy foods but they were also concerned about differentiated access and increased inequalities. The supermarket could also put some local neighborhood food retailers out of business. Ultimately, some viewed the mall as improving the economy of the local populations, while others and particularly local community stakeholders did not think that the local population would benefit, in monetary terms, as much from the mall.

Concerning impacts on the local population and environmental health, both regulatory and local community stakeholders seemed to agree that the mall and supermarkets would fuel both NCDs, through access to unhealthy food, and environmental pollution, from increased vehicular traffic, among other ills in the local community. They also agreed that other social ills such as crime would increase because of the increased number of people visiting the mall and supermarkets.

There was evidence of substantial multi-sectoral responsibility in issues related to diet and health. Non-health sector stakeholders raised a range of concerns that have either direct or indirect impacts on health or the social determinants of health in the same way that health sector stakeholders also raised wider issues outside of their immediate remit such as local transport business opportunities. This has important implications for the campaign against the spread of NCDs since all stakeholders who are important influencers in the community were able to carry the same message to the community and create a synergy that energizes the fight to reduce the incidences of NCDs locally. It was clear that the non-health stakeholders desired more information about the risk factors associated with NCDs. It is useful to conduct workshops to give stakeholders information about the importance of research evidence for policy decision-making, especially in the health field.

Contrary to current literature from HICs about the impact of supermarkets on local populations, stakeholders we interviewed thought the mall and supermarkets would deepen dietary inequalities in Western Kenya when wealthier local residents consumed unhealthy food purchased from these outlets, while poorer residents stuck by their local food vendors who sell less processed food at fairer prices. This differs from high-income countries in which healthy foods are typically viewed as more expensive than unhealthy foods (21, 22).

### Comparison with other related literature

Adopting a socio-ecological framework for understanding stakeholder perceptions allowed us to collect more nuanced information. Nsamenang (34) applies an Afro-centric lens to interpret the socio-ecological model of human behavior in the field of child development. He stresses that people use their culture (cultural constructions) to make sense of their environment. What may not seem important to an “outsider” could well be the point at which an intervention such as promoting healthy dietary practices may be successful. In this instance, local community stakeholders were more of “insiders” in local communities than regulatory stakeholders. They know the local cultures and thus could easily appeal to and persuade them to be wary of unhealthy foods sold in supermarkets. This is comparable to findings by, McCormack et al. (35) from their systematic review, discussing health literacy in the USA when they note that the socio-ecological model allows for the interventions to improve patient literacy at various levels of action in the community as presented by the model. Additionally, Hull et al. (36) in their narrative systematic review aimed at collating, summarizing, and synthesizing evidence related to physical activity interventions aimed at young female subjects (14–25 years) in the United Kingdom, explore measures used to evaluate the impacts of the interventions and recommend the good practice to future similar research as best conducted using an ecological model. Indeed, this study has shown that local community stakeholders are very important forces that shape thinking among community members thus apt drivers for health interventions.

Most health impact assessment (HIA) literature is based on quantitative studies thus not focusing much on the value of evidence that can be generated by qualitative and participatory studies. In a systematic review of available studies on Health Impact Assessment, Thondoo et al. (37) noted that only 12% of this literature was based on participatory approaches and 72% was based on the quantitative methodology. Our study is based on both qualitative and participatory approaches to generating evidence and thus makes an important contribution by advancing evidence in this area.

### Implications for policy and practice

A multi-sectoral approach to combating NCDs aims to work by harnessing skills and abilities found in various sectors of the country's economy and focusing this resultant synergy to fight NCDs (28). In our study, stakeholders across multiple sectors had suggestions about how to engage in NCD prevention in the community, particularly regarding the availability of unhealthy food.

Local community stakeholders were concerned about the community's physical and mental health. In particular, faith leaders and organizations are seen as highly persuasive to the estimated 70% of Kenyans who self-describe as being religious (38). They are key actors engaged in passing messages of healthy nutrition and diets to their congregations and letting the food outlet owners know what the faithful wish them to stock. As noted by Nsamenang (34) individuals act within a cultural backdrop, so even if the cultural stakeholders do not take an overt part in licensing business premises for operation, they tacitly decide the success of such ventures. Indeed, it is a requirement in Kenya that before a project of the magnitude of the Lake Basin Mall is developed, officials must invite the public to give their input in a public participation workshop. At this time, institutions of higher learning, faith, environment, and other community stakeholders get a chance to contribute their views aimed at improving the proposed project, especially on its impact on the environment and local communities. However, such forums are at times not held, or the views collected are not used to improve target projects, because of the corruption often associated with such projects. The Lake Basin Mall is no exception and has had its share of corruption scandals according to a 2019 report by EACC (31).

### Research implications of the study

Further work should include an assessment of the true impact of the Lake Basin Mall on the health of the surrounding population and local business opportunities as is being completed in our natural experimental study. Evaluation of the apparent early multi-sectoral stakeholder interest in health including any evidence of an impact on policy or decision making would also be of value alongside wider work exploring the stakeholder perspectives on topics of public health importance, in particular in relation to NCDs.

### Strengths and limitations of the study

This is the first exploration of multi-sectoral stakeholder perspectives on a new mall and supermarket in Western Kenya. Previous studies disproportionately dwelt on the health impacts of living near a supermarket (24–26) for local communities. This study goes further and provides a rare in-depth insight into stakeholder considerations and potential impacts of the foodscape on diet and health and by so doing exposes important intervention avenues and possibilities in the campaign against NCDs and environmental degradation in local communities. As far as we know, this is the first of this type of study; a natural experiment surveying the impact of a new mall and supermarket on the diets and environment of local populations. This study is then an important entry point for such studies focusing on the causes of Kenyans' changing dietary behaviors, emerging health and wellbeing experiences, and possible ways to intervene. The study was limited to the Western part of Kenya and further to Kisumu and Homa Baytowns in this expansive region. Despite this limitation, the findings easily speak to communities that have access to new developments such as the Lake Basin Mall in Kisumu and supermarkets in Homa Bay.

## Conclusion

We explored stakeholder perspectives on the impacts of new and existing supermarkets on local diets, population health, and wellbeing. Our key finding was the evidence of a multi-sectoral approach with a widely held interest in the potential impacts of the malls and supermarkets on the health and wellbeing of the local population. We conclude that both the regulatory and local community stakeholders understand to varying degrees the health impacts of the newly developed Lake Basin Mall in Kisumu and supermarkets in Homa Bay. Whereas regulatory stakeholders are formally trained in the area in which they identify their expertise, the local community stakeholders use a range of sources of knowledge from across their environment. Indeed, as local community influencers, they already shape opinions about healthy diets and behavior albeit their rudimentary knowledge of the epidemiology of NCDs. These two groups of stakeholders present an accessible opportunity for countries in LMICs, such as Kenya, with an ever-present unmet need for healthcare staff, to develop and sustain campaigns against NCDs in local communities if offered more information about managing NCDs and other health messages. Additionally, urban development initiatives, which are often considered markers of “progress” clearly need to consider health impacts, and one important way to assess this impact is to gather perceptions from community members. Our findings have relevance for other rapidly urbanizing contexts characterized by increased NCDs coupled with resource constraints which makes the prevention of disease and access to healthy environments, necessary for health and wellbeing, critical. In such rapidly urbanizing contexts, the importance of both health impact and environmental impact assessments as cities develop to ensure opportunities to create health equitably are not missed and can never be overstated.

## Data availability statement

The raw data supporting the conclusions of this article will be made available by the authors, without undue reservation.

## Ethics statement

The studies involving human participants were reviewed and approved by Scientific Ethics Review Unit (SERU) Kenya Medical Research Institute, Kenya. The patients/participants provided their written informed consent to participate in this study.

## Author contributions

PW led conceptualization and development of manuscript. CO, VW, RM, EM, LF, ET-M, and OF contributed in developing manuscript and approving it for submission. All authors contributed to the article and approved the submitted version.

## Funding

This research was funded by the National Institute for Health Research (NIHR) (16/137/64) using UK aid from the UK Government to support global health research.

## Conflict of interest

The authors declare that the research was conducted in the absence of any commercial or financial relationships that could be construed as a potential conflict of interest.

## Publisher's note

All claims expressed in this article are solely those of the authors and do not necessarily represent those of their affiliated organizations, or those of the publisher, the editors and the reviewers. Any product that may be evaluated in this article, or claim that may be made by its manufacturer, is not guaranteed or endorsed by the publisher.

## Author disclaimer

The views expressed in this publication are those of the author(s) and not necessarily those of the NIHR or the UK Department of Health and Social Care.
